# Visualizing Relaxation in Wearables: Multi-Domain Feature Fusion of HRV Using Fuzzy Recurrence Plots

**DOI:** 10.3390/s25134210

**Published:** 2025-07-06

**Authors:** Puneet Arya, Mandeep Singh, Mandeep Singh

**Affiliations:** Department of Electrical and Instrumentation Engineering, Thapar Institute of Engineering and Technology, Patiala 147004, India; mdsingh@thapar.edu (M.S.); mandeep@thapar.edu (M.S.)

**Keywords:** relaxation, slow-paced breathing, heart-rate variability, fuzzy recurrence plot, autonomic nervous system

## Abstract

**Highlights:**

**What are the main findings?**

- A novel method was developed to convert HRV time series into textured images using fuzzy recurrence plots (FRPs) based on fuzzy set theory.
- The model achieved 96.6% classification accuracy for relaxation states using only three selected features across multiple domains.

**What are the implications of the main findings?**

- The model enables both visual and automated interpretation of physiological changes during relaxation, enhancing transparency and user engagement.
- The model is suitable for real-time integration into low-power wearable devices for stress monitoring and biofeedback.

**Abstract:**

Traditional relaxation techniques such as meditation and slow breathing often rely on subjective self-assessment, making it difficult to objectively monitor physiological changes. Electrocardiograms (ECG), which are commonly used by clinicians, provide one-dimensional signals to interpret cardiovascular activity. In this study, we introduce a visual interpretation framework that transforms heart rate variability (HRV) time series into fuzzy recurrence plots (FRPs). Unlike ECGs’ linear traces, FRPs are two-dimensional images that reveal distinctive textural patterns corresponding to autonomic changes. These visually rich patterns make it easier for even non-experts with minimal training to track changes in relaxation states. To enable automated detection, we propose a multi-domain feature fusion framework suitable for wearable systems. HRV data were collected from 60 participants during spontaneous and slow-paced breathing sessions. Features were extracted from five domains: time, frequency, non-linear, geometric, and image-based. Feature selection was performed using the Fisher discriminant ratio, correlation filtering, and greedy search. Among six evaluated classifiers, support vector machine (SVM) achieved the highest performance, with 96.6% accuracy and 100% specificity using only three selected features. Our approach offers both human-interpretable visual feedback through FRP and accurate automated detection, making it highly promising for objectively monitoring real-time stress and developing biofeedback systems in wearable devices.

## 1. Introduction

Stress is a growing concern in modern society, contributing to various psychological and physiological disorders. The autonomic nervous system (ANS) is responsible for regulating hemostasis through two opposite mechanisms, namely the sympathetic nervous system (SNS) and the parasympathetic nervous system (PNS). The SNS is triggered by a stress-inducing modulator called a stressor [1], while the PNS is triggered by a relaxation-inducing modulator called a relaxer [2]. In recent years, diverse relaxation-inducing modulators have been investigated. These modulators include guided imagery, music, olfactory stimulation (fragrance), exercise, and slow-paced breathing [3,4,5,6,7,8].

Among these, slow-paced breathing in the frequency range 0.05–0.15 Hz (3–9 breaths/min) has been shown to enhance parasympathetic activity and reduce stress levels [7,9,10,11,12]. Meditative practices have long incorporated slow breathing techniques to induce relaxation by modulating the heart’s activity [13], resulting in respiratory sinus arrhythmia (RSA). This is a phenomenon where the heart rate (HR) increases during inhalation and decreases during exhalation [9]. These changes can be quantitatively assessed using heart rate variability (HRV), a non-invasive measure of ANS function derived from the time difference between successive R peaks in an electrocardiogram (ECG) signal [14,15,16].

In previous studies, researchers have assessed the changes in HRV through time-domain, graphical, frequency-domain, and non-linear methods, each contributing unique insights into autonomic nervous system (ANS) dynamics [17]. The time-domain methods are directly applied to the RR interval trend line. They are simple to compute and provide statistical parameters. The frequency-domain method involves a power spectrum estimate of the RR interval time series. The method decomposes the signal into the frequency components, namely very low frequency (VLF), low frequency (LF), high frequency (HF), and total power (TP). The LF band power is suggested to be produced by both the PNS and SNS and is associated with BP regulation. The HF band is linked with parasympathetic activity [18,19].

The time domain often suffers from an averaging effect and fails to capture the interaction between the ANS and the heart entirely. The method is sensitive to the non-stationary nature of HRV time series during slow-paced breathing, limiting their reliability for population-level analysis. The interpretation of frequency-domain methods is subjective and requires information about the psychological state. The LF band power increases both in slow-paced breathing and during stress events. Therefore, non-linear methods like entropy are used. Entropy is the measurement of the regularity and complexity of a time series. However, entropy depends on the optimal value of the hyperparameter for analysis.

We propose a novel method that converts HRV time series into images using fuzzy recurrence plots (FRPs) to analyze repeating patterns. This approach offers two key benefits: it visually highlights changes in autonomic activity, making it easier for even non-experts to interpret, and it provides rich texture features for further automatic classification. FRPs generate greyscale texture images from time-series data and have been effectively used in analyzing signals from photoplethysmography (PPG) and gait [20,21,22]. Like these signals, HRV exhibits repeating patterns over time. These patterns vary between spontaneous and slow-paced breathing, and FRPs help visualize both regular (periodic) and irregular (aperiodic) changes. During slow-paced breathing, the heart rate changes within each breath appear clearly as structured textures in the FRP images, capturing important physiological dynamics. These texture signatures yield novel physiological insights into autonomic nervous system (ANS) modulation, particularly respiratory sinus arrhythmia (RSA), by highlighting how the heart adapts dynamically during each breathing cycle. This not only enables differentiation between breathing states, but also serves as a potential tool for clinicians to observe real-time physiological responses to paced respiration, a known marker of parasympathetic activation and overall cardiovascular health.

Previous studies have demonstrated the transformation of ECG signals into binary images for arrhythmia detection using deep convolutional neural networks, such as via EfficientNet, AlexNet, VGG-16, and Inception-V3 architectures [23,24,25]. These previous works primarily target pathological contexts, focusing on morphological variations within the ECG signal to identify ventricular tachyarrhythmias [26]. In contrast, our work differs in both objective and method. We do not focus on detecting cardiac pathologies. Instead, we study physiological modulation in healthy individuals. Specifically, we examine the autonomic response during slow-paced breathing. In this setting, there are minimal or no morphological changes in the ECG. Rather than using ECG morphology, we extract RR interval time series. These RR intervals reflect the balance between sympathetic and parasympathetic activity. As a result, HRV serves as a sensitive marker of autonomic function.

Crucially, we use fuzzy recurrence plots (FRP) instead of threshold-based binary recurrence plots. Binary recurrence plots may fail to capture the subtle, nonstationary, and quasi-periodic nature of HRV. They rely on fixed thresholds, which may not handle individual variability well. In contrast, FRP uses adaptive fuzzy clustering. It assigns membership values to data points, creating richer texture-based images. This approach better preserves the temporal complexity caused by respiration-induced HRV changes. The FRP images are then used for feature extraction. This allows us to capture fine details of physiological modulation related to breathing patterns in healthy individuals.

In this study, we explore these visual transformations in HRV during slow-paced breathing and introduce a multi-domain feature fusion framework for relaxation state detection. This framework integrates time-domain, frequency-domain, non-linear, and texture-based features, enabling a comprehensive and discriminative characterization of psychophysiological states. Given the high dimensionality of the extracted features, we employ a feature selection strategy using the Fisher discriminant ratio (FDR), correlation analysis, and greedy search to identify the most relevant subset for classification. We further evaluate the effectiveness of our approach using six machine learning classifiers, demonstrating that FRP features significantly improve classification accuracy. The proposed system provides real-time, objective feedback on relaxation states and is well-suited for integration into wearable health devices, such as smartwatches [27]. By supporting time-sensitive biofeedback mechanisms, this approach holds strong potential for improving stress management and psychophysiological state monitoring in everyday settings.

## 2. Materials and Methods

### 2.1. Participants

Sixty volunteers aged between 18 and 31 years participated in this study. The study is conducted following the Declaration of Helsinki. The ethical committee of Thapar Institute of Engineering and Technology approved the study. The participants were informed about the experiment’s protocol and signed an informed consent form. All the participants were healthy and declared their mental and physical well-being. The data acquisition methodology was non-invasive, and the study involved young and healthy subjects; hence, no medical supervision was required. All the data analysis and reporting were performed anonymously. All the participants were given the option to withdraw from the study.

### 2.2. Experimental Protocol

In a quiet environment, the participants were seated in a comfortable chair for data acquisition. They were instructed to sit straight in the chair and breathe comfortably. The experimental protocol was 10 min long and had two phases of 5 min each. An ECG signal was acquired during each phase. Furthermore, the participants were instructed to avoid hand and leg movement during the data acquisition to avoid motion artefacts.

**Figure 1:**
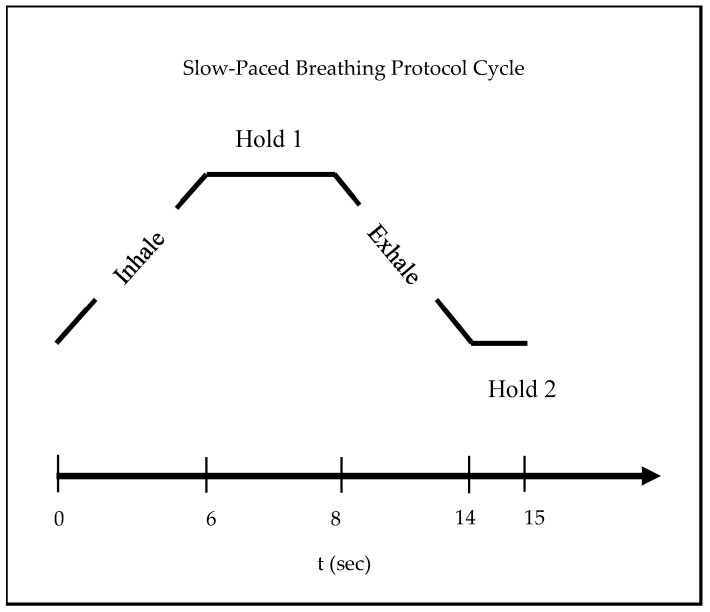
Experimental protocol depicting slow-paced breathing phase.

The first data acquisition phase was the rest phase, during which the participants were instructed to breathe normally with closed eyes. The second phase was a slow-paced breathing task. An audio recording was played using two tones to assist the participants in following the slow-paced breathing protocol. The participants were instructed to inhale during tone 1 and exhale during tone 2. There was a pause of 2 s at the end of inhalation and a pause of 1 s at the end of exhalation. The duration of inhalation and exhalation was 6 s. The total duration of a complete breathing cycle was 15 s. Hence, this made the respiration frequency 4 breaths per minute. Figure 1 shows the slow-paced breathing experimental protocol used in the study.

### 2.3. Data Acquisition

The ECG was acquired using a custom-designed data acquisition system. The non-invasive acquisition method involved standard pre-gelled electrodes (Ag–AgCl). The electrodes were placed on the inner sides of two forearms and the right ankle as per lead 2 configuration. The ECG signal was pre-processed using a notch filter and a Savitzky–Golay filter to obtain a smooth signal [28]. The acquisition system detects the R peak and the location in the ECG signal based on the Pan–Tomkins algorithm [29].

The time difference between the two successive R peaks (heart rate variability) was obtained and stored. The signal is visually inspected for abrupt changes in HRV time series. The HRV time series was further pre-processed to remove all the outliers due to any skipped heartbeats and false detection. A threshold range-based criteria was used for removing outliers. If a beat laid outside (333–2000 ms), it was removed [30].

### 2.4. Analysis of HRV Time Series

The HRV time series was assessed using five methods: time-domain, frequency-domain, graphical, entropy, and image-based. The image-based method used the fuzzy recurrence plot method to convert the HRV time series to an FRP image. Then, grey-level co-variance matrix features (FRP features) were computed for each FRP image.

#### 2.4.1. Time-Domain, Frequency-Domain, and Graphical HRV Features

Time-domain, frequency-domain, and graphical HRV features were extracted from an open-source HRV analysis software SinusCor, version 1.0.0 [31]. The software can accept raw ECG and HRV time series in a text file. It can compute time-domain, frequency-domain features, and graphical features. The signal was resampled at 4 Hz using cubic spline interpolation to obtain a signal with equidistant sampled time series for frequency-domain analysis. The details of the features are tabulated in Table S1.

#### 2.4.2. Non-Linear HRV Features Using Entropy

This study used five entropy-based features, namely approximate entropy, sample entropy, fuzzy entropy, amplitude-aware permutation entropy, and bubble entropy, to capture the complexity of the HRV time series during spontaneous breathing and slow-paced breathing inducing relaxation. An open-source software CEPS (pipeline_v2) was used to compute entropy measures for the acquired HRV time series [32]. Table 1 shows the hyperparameters used for each entropy method.

#### 2.4.3. Time-Series to Image Conversion Using FRP

A fuzzy recurrence plot (FRP) describes the relationship between two data points in time-series signals based on a fuzzy relationship. The FRP is a modification of the recurrence plot (RP). In RP, a *NxN* matrix *R* is created. Each element of a matrix is denoted as *R*(*i, j*). The value *R*(*i, j*) is the relationship between two data points (*x_i_, x_j_*), and is quantified using the threshold technique. To illustrate, if the difference between the two data points is above the threshold value (*ε*), then 1 is assigned to the relationship using the Heaviside step function (H); if not, 0 is assigned, as shown in Equation (1). Hence, in RP, a binary image is created. An optimal threshold value is required to obtain visually distinguishable RP binary images for different classes of signals. Then, features are extracted using recurrence quantification analysis. The RP method is also used in biomedical signal classification involving ECG, respiration, and HRV time series [33,34,35]. *X* is a time-series signal with *N* data samples, *X* = [*x*_1_, *x*_2_, *x*_3_, …, *x_n_*]

(1) R(i, j)=H(ε−‖xi−xj‖)

where *x_i_* and *x_j_* are *ith* and *jth* sample, *ε* denotes the threshold value, *H* is the Heaviside step function, *R*(*i, j*) represents the value given to the relationship between the pair of states (*x_i_*, *x_j_*) of the recurrence plot matrix *R*.

Similarly to RP, a *NxN* matrix is obtained in FRP. In FRP, the value assigned to relation is in the range 0 to 1. On one hand, RP used strict boundaries leading to discontinuity, therefore a black and white image is created. On the other hand, FRP is continuous. Hence, fuzzy recurrence plots convert a time-series signal into a greyscale textured image. Therefore, the techniques involving feature extraction from greyscale textured images could be applied effectively, like GLCM. Additionally, the computation of the pixel values in FRP is based on fuzzy membership; hence, the computation of the optimal threshold value is not required.

Image formation using FRP is a two-step process. The first step is to find optimal fuzzy cluster centres. The second step is to use fuzzy relationship properties to obtain pixel values for an FRP image. The algorithm is briefly described here.

Let *X* = [*x*] represent an array of samples of a time-series signal, and *V* = {*v*} be the set of fuzzy clusters (FC). Each FC is represented by its cluster centre *z*, and the dimension of each cluster centre is the number of columns of *X*. There are c clusters, and the number of clusters is provided by the user. Let *Z* be a list of the cluster centres. Let *S* be the fuzzy relationship between *X* and *V*. To illustrate, each sample value (*x_i_*) has a relation with each cluster (*v_j_*), which is quantified by the degree of membership *μ_ij_*.

The cluster centres are optimized using the fuzzy C-means clustering (FCM) algorithm briefly discussed here.

Objective function of FCM algorithm:

The minimization of objective function J involves an iterative process of updating the values of *U* and *Z*. *U* is a matrix of fuzzy partition, *U* = [*μ_ij_*] where *i* = 1, …, *N* and *j* = 1, …, *c*, and *μ_ij_* membership value of *ith* data sample for *jth* cluster. For a given data point, *x_i_*, the sum of membership value for all clusters is one. *Z* is a list of the cluster centre, *Z* = [*z*_1_, *z*_2_, …, *z_c_*].

(2) $$J\left(U,Z\right)=\sum_{i=1}^{N}\sum_{j=1}^{c}{\left(\mu_{ij}\right)}^{m}\;{\left[d\left(x_{i},z_{j}\right)\right]}^{2}$$

where *N* is the data sample length, *c* is the number of clusters, 1 < *c* < *N*, *m ϵ* [1, ∞) is the fuzzy weighting exponent, *μ_ij_* is the membership value of *ith* data sample for *jth* cluster, *d*(*x_i_, z_j_*) is the Euclidean distance computed between *ith* data sample and *jth* cluster centre. The membership value and cluster centres are updated using Equations (3) and (4).

(3) μik=1∑j=1cdxi,zkdxi,zj2m−1, 1≤k≤c

where *i* = 1, …, *N*, *j* = 1, …, *c* and *Z* is defined as a vector (*z*_1_, *z*_2_, *…, z_c_*) where *z_j_* is and *j* = 1, …, *C*

(4) $$z_{j}=\frac{\sum_{i=1}^{N}{\left(\mu_{ij}\right)}^{m}x_{i}}{\sum_{i=1}^{N}{\left(\mu_{ij}\right)}^{m}},\forall_{j}$$

The iterative process starts with the first random initialization of *U*. Then, *Z* cluster centres are computed using Equation (4). Then J is computed, and *U* is updated based on Equation (2). The convergence criteria for the objective function are defined as $\left|U^{t}-\right.\left.U^{t+1}\right|\leq \epsilon$, where the level of accuracy (*ϵ*) is set at 0.00001 after computing the optimal cluster centres and membership value. In other words, for each data point of the time-series signal, the fuzzy relationship is obtained. This fuzzy relationship is used to obtain the fuzzy relationship between two data points of time-series signals. The obtained value is the pixel value for FRP image. The three properties, namely, reflexivity, symmetry, and transitivity (max-min operator) of fuzzy sets, are used. It is noteworthy to mention two observations; first, the FRP image has a constant diagonal value based on the reflexivity property (Equation (5)). Second, the image is symmetric about the diagonal based on the symmetry property (Equation (6)).

- Reflexivity:

(5) $$\mu \left(x,x\right)=1,\;\forall x\;\in X$$

- Symmetry:

(6) $$\mu \left(x,\upsilon \right)=\mu \left(\upsilon ,x\right),\;\forall \;x\in X,\;\forall \;v\in V$$

- Transitivity:

(7) $$\mu \left(x,z\right)=\vee_{\upsilon }\left[\mu \left(x,\upsilon \right)\wedge \mu \left(\upsilon ,z\right)\right],\text{ \#xA0;}\forall \;x\;\in Χ\;,\;\;\forall \;v\;\in \;Ζ$$

where the symbol ∨ is a max operator, ∧ is a min operator, and the equation is a max-min composition.

Since the values assigned to the relationship between two data points range from 0 to 1, the FRP (fuzzy recurrence plot) image is represented in greyscale. This means that the FRP image can visually represent HRV (heart rate variability) time-series data. Different HRV time series, such as those recorded during spontaneous versus slow-paced breathing, exhibit distinct textures in the FRP image.

**Figure 2:**
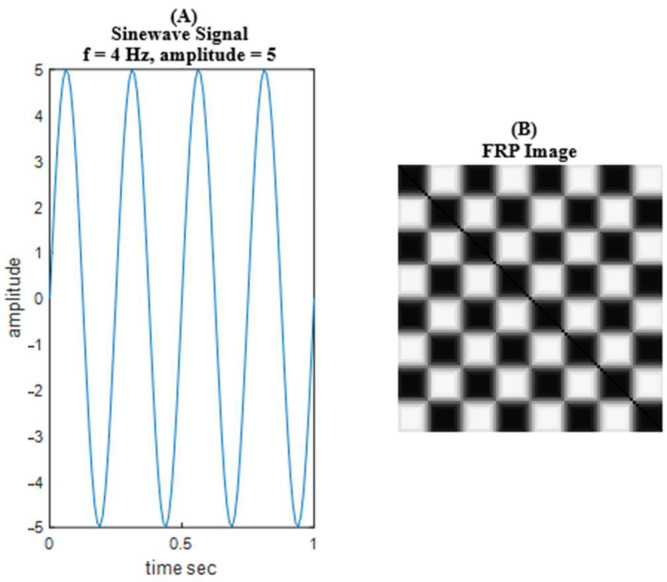
FRP image of a sine wave signal.

For example, Figure 2 illustrates an FRP image of a sine wave signal with a frequency of 4 Hz, an amplitude of 5, a duration of 1 *s*, and a sampling rate of 100 Hz. In this FRP image, you can observe a constant diagonal line with symmetry about this diagonal. This symmetry is due to the periodic nature of the sine wave signal, which results in a repeating pattern in the greyscale levels of the image.

The GLCM method was proposed by Haralick in 1973 and is widely used in texture analysis. GLCM represents the relationship between neighbouring pixels and is defined as a statistical joint probability of two pixels [36]. In other words, the GLCM value expresses the probability of pixel *i* and pixel *j* having the same intensity. First, the image is transformed into discrete grey levels. Generally, 8 discrete levels are used, and an 8 × 8 GLCM matrix is computed. The GLCM is constructed using two parameters, namely the relative distance (*d*) and orientation (*ϕ*). The relative distance is measured in pixel number, and orientation is fixed as one of the four directions (horizontal—0°, diagonal—45°, vertical—90°, and anti-diagonal—135°) [37]. In this research, the parameter distance was set to be 1, and the orientation was horizontal—0°.

A GLCM *G_d_* (*k, l*) can be generated at distance d and along direction *ϕ* as:

(8) $$G_{d}\left(k,l\right)=\sum_{i}\sum_{j}\sum_{\theta }\delta \left[k,p\left(i,j\right)\right]\delta \left[l,p\left(\left(i,j\right)+d\theta \right)\right]$$

where *k, l* are grey values, *p*(*i, j*) is the grey value of a pixel at position (*i, j*), *p*((*i, j*) *+ dθ*) is the grey value of a pixel at a position d units away and at an orientation defined by the direction *θ* about *p*(*i, j*). The Kronecker delta, *δ*(*a*, *b*) takes a value of 1 if *a = b*, or else 0 when *a ≠ b*.

This study computed 19 different GLCM features along horizontal orientation from each FRP image. The features describe the statistical and textural properties of the image. They have great capability in exploring and quantifying textural changes. These features have been widely used in biomedical signal analysis, including the classification of iris images for diabetic patients, gait dynamics assessment, and liver pathological assessment [21,38,39].

The features used in this study include autocorrelation, contrast, correlation, cluster prominence, cluster shade, dissimilarity, energy, entropy, homogeneity, maximum probability, sum of squares variance, sum average, sum variance, sum entropy, difference variance, difference entropy, information measure of correlation1, information measure of correlation2, and inverse difference. The details of these features are tabulated in Table S2.

It is noteworthy to mention here that we observed that it takes approximately 300 milliseconds to generate the FRP image and GLCM feature computation on a ASUS Vivobook manufactured by ASUSTeK COMPUTER INC, Chongqing China, with a configuration of Intel’s i5-12500H processor (2.5 GHz) and 16 GB RAM. The computational time is about 0.001% of the total data acquisition time of 5 min.

### 2.5. Statistical Analysis

The involvement of people or animals in the biomedical data collection process leads to small sample sizes; therefore, the data do not follow a normal distribution. In this study, the Kolmogorov–Smirnov test indicated that the data was not normal. Therefore, we used the Wilcoxon signed-rank test proposed by Frank Wilcoxon [40]. The test is a non-parametric alternative to the paired Student’s *t*-test. The Wilcoxon signed-rank test is used to determine whether the population mean ranks of the samples differ [41]. Here, the test statistic, W, is the sum of the ranks of positive differences between the observations in the two samples. The features with *p*-value < 0.05 are considered to be statistically significant. This study used MATLAB 2019a’s built-in function for the Kolmogorov–Smirnov test and the Wilcoxon signed-rank test for evaluation.

In addition to statistical significance, measuring the effect size is crucial to quantify the magnitude of change between paired conditions. Since the Wilcoxon signed-rank test is a non-parametric alternative to the paired Student’s *t*-test, it requires a non-parametric measure of effect size. In this study, we used rank–biserial correlation (RBC) to estimate effect sizes. RBC is an appropriate and robust metric for non-parametric paired data, especially when assumptions of normality are violated. It quantifies the difference in ranks between two related groups and provides interpretable values ranging from −1 to +1. Table S3 provides the interpretation of RBC values, where values closer to ±1 indicate stronger and more consistent effects between the paired conditions. Unlike Cohen’s *d*, which assumes normally distributed data and equal variances, RBC aligns well with the assumptions and logic of the Wilcoxon test. Therefore, RBC was selected as a compatible and informative effect size measure for this analysis. The RBC values were computed following the methodology described by Kerby [42].

### 2.6. Feature Selection and Reduction Method

It is possible to use the classifier to use all the 39 features of time-domain, frequency-domain, graphical, non-linear, and FRP features. However, this may decrease the computational and discriminatory performance of the classifier.

In this study, first, a *z*-score-based data standardization technique was applied. The technique transforms the data to a common scale with a mean of around zero and a standard deviation of one. Afterwards, Fisher’s discriminant ratio (FDR) and correlation matrix-based dimensionality reduction methods were used. Additionally, many features likely become relevant; therefore, a greedy search-based method was applied to relevant and non-redundant feature sets to obtain a smaller feature subset. The employed methods are briefly explained here.

- Fisher’s Discriminant Ratio

The Fisher linear discriminant method is widely used in binary data classification feature selection. FDR is used to quantify a feature’s discriminatory power between two classes.

(9) $$FDR=\frac{{\left(\mu_{1}-\mu_{2}\right)}^{2}}{{\sigma_{1}}^{2}+{\sigma_{2}}^{2}}$$

where *μ*_1_ and *μ*_2_ are mean values, and *σ*_1_ and *σ*_2_ are variances of a feature vector of the two classes (1 and 2).

The FDR value is higher for features with large differences between the means of the two classes and smaller variances (within-class scatters) in each class [39]. So, the features are sorted in descending order based on their FDR values for further processing.

- Correlation Matrix

The FDR method sorted the features, and then the correlation matrix method was applied to the obtained non-redundant features [39]. The threshold value for feature rejection was set to be 0.9.

- Classifier Subset Evaluator (CSE)

The CSE evaluates the subsets of features by using a classifier on the training data to find the best subset of features. Here, we employed a greedy stepwise search-based evaluator. A greedy stepwise search starts with an empty set, then features are selected using forward selection, and useless features are eliminated using backward selection. In the search process, new features are added to the subset, and performance is evaluated. The current subset is modified when the classification performance improves, generating a best-feature subset. The algorithm continues until the newly generated subset does not surpass the best current subset [43].

### 2.7. Classifier and Performance Parameters for Relaxation Detection

In our experiments, we chose six machine learning-based classifiers for the binary classification mentioned in Table 2. All classifiers are available in the WEKA machine learning platform. WEKA version 3.8.3 is an open-source platform with a GNU General Public Licence [44,45].

A 10-fold cross-validation scheme was adopted to estimate classification performance [46]. Then, the classification performance was evaluated with the following four measures using the confusion matrix: true positive (*TP*), false positive (*FP*), true negative (*TN*), and false negative (*FN*). The sensitivity, specificity, accuracy, and *AUC* were computed to further demonstrate the classifier’s discriminatory performance. These parameters are frequently used in medical diagnostics to categorize the positive and negative patients of a disease [18,39]. Sensitivity indicates the strength of the classifier’s performance in detecting disease. Specificity indicates the strength of the classifier’s performance in detecting patients who do not have the disease.

The *AUC* is the area under the receiver operating characteristics (*ROC*) curve. The *ROC* curve plots the true-positive rate (sensitivity) on the *y*-axis and the false-positive rate (1-specificity) on the *x*-axis at various threshold settings. The *ROC* curve is useful in visualizing and evaluating the performance of classifier models. The curve shows the relative trade-offs between the model’s benefits (true positives) and costs (false positives).

In addition to cross-validation, a learning curve analysis was performed to study how training size influences classification performance. This involved a holdout-based evaluation where the dataset was split into multiple training/test partitions, ranging from 10% to 90% test set proportions (i.e., 90% to 10% training sizes). For each split, classification was performed using selected machine learning models to observe their behaviour across different data volumes.

## 3. Results

**Figure 3:**
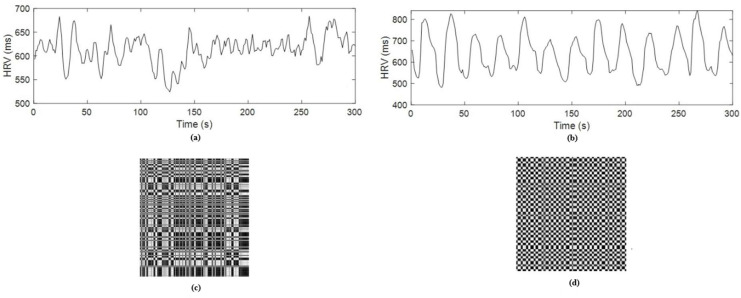
HRV time series: (**a**) spontaneous breathing, (**b**) slow-paced breathing and FRP images, (**c**) spontaneous breathing, (**d**) slow-paced breathing.

In this study, two sets of HRV time-series signals were acquired. The first was a baseline HRV time series of 5 min duration and was obtained during spontaneous breathing, as shown in Figure 3a. The second HRV time series was obtained under slow-paced breathing at four breaths per minute with a ratio of inspiration time to expiration time of 1:1, as shown in Figure 3b.

### 3.1. Multi-Domain Analysis of HRV Features

The analysis of the HRV was conducted using HRV features based on time-domain, frequency-domain, graphical, non-linear (entropy), and image-based methods. A total of 15 HRV features belonging to time-domain, frequency-domain, and graphical methods were obtained from open-source HRV analysis software SinusCor version 1.1. The mean and standard deviation of the time-domain, frequency-domain, and graphical-domain features are tabulated in Table S4. Five entropy-based non-linear features were obtained from open-source software CEPS. The mean and standard deviation of entropy-based features are tabulated in Table S5. HRV time series were converted into FRP images to obtain 19 FRP features. The mean and standard deviation of the FRP features is tabulated in Table S6.

Figure 3c,d display the FRP images of HRV time series during spontaneous and slow-paced breathing. The texture differences between the two images are visually significant, with slow-paced breathing showing more prominent patterns than spontaneous breathing. These visual distinctions suggest that texture analysis of the FRP images could play a key role in classifying the two states. We computed 19 GLCM features for each FRP image to capture these texture variations. Given the visible differences, this method shows great potential for improving classification accuracy between spontaneous and slow-paced breathing. Using GLCM-based texture features enhances the discriminatory power of the FRP image-based method for HRV analysis. Notably, T.D. Pham initially proposed the FRP image-based method, which was highly successful in classifying gait signals with 100% accuracy [21].

To evaluate the paired effect of features during spontaneous breathing and slow-paced breathing, we conducted a Wilcoxon signed-rank test and calculated the rank–biserial correlation (RBC) as a non-parametric measure of effect size. The RBC values range from −1 to +1, where values closer to either extreme denote stronger and more consistent effects. Several features demonstrated exceptionally strong and consistent changes between conditions. For example, features such as SampEn_DS, entro, and LF had perfect or near-perfect positive RBC values (RBC ≈ +1), indicating substantial increases following the slow-paced breathing. Conversely, strong negative RBC values were observed for features like dissi, denth, and ApEn, reflecting pronounced decreases. Some features, such as MeanHR and HF, showed minimal changes (|RBC| < 0.1), indicating negligible paired effects. This analysis highlights the most responsive features for downstream modelling and interpretation. A complete summary of RBC-based effect direction and strength is provided in Table S7, offering a transparent and interpretable overview across all domains of extracted features.

### 3.2. Classification of HRV Time Series

After extracting features from all five methods (time-domain, frequency-domain, graphical, non-linear, and time-series-to-image conversion), we applied the Fisher discrimination ratio (FDR > 0.95) for feature selection. Table 3 shows that thirteen relevant features were selected after this stage. Notably, eight features come from the FRP image-based method, indicating its discriminatory solid power in distinguishing spontaneous and slow-paced breathing states. Three features were from the frequency domain, one from the non-linear method, and one from the graphical domain.

Following feature selection, we used a correlation matrix technique to remove redundant features, further refining the feature set. Table 4 presents the final set of five non-redundant features. This step underscores the high correlation among FRP features, which, although powerful in discrimination, need to be reduced for practical applications. It is worth emphasizing that FRP features had the highest FDR values, with cluster prominence emerging as particularly strong compared to time-domain, frequency-domain, and graphical features. Ultimately, only one FRP feature was retained in the final set due to the high correlation, highlighting the strength and redundancy of features derived from the FRP image-based method. This supports our earlier observations about the effectiveness of FRP image-based feature extraction for HRV analysis.

**Figure 4:**
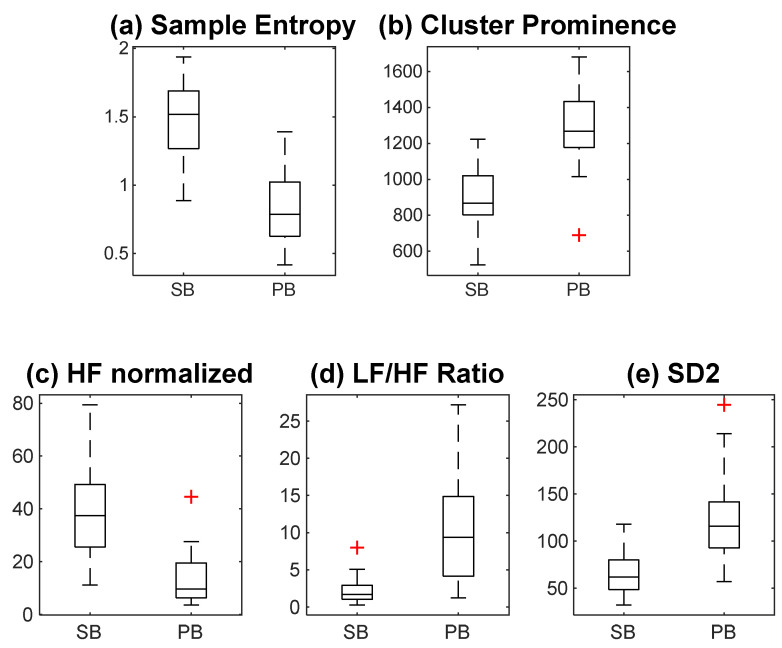
Box plot of five relevant and non-redundant features. The box plot displays the distribution of values for each selected feature. The Red plus signs (+) indicate outliers beyond the whisker range.

Figure 4 shows the box plot of relevant and non-redundant features. Figure 4a shows non-linear entropy features. Figure 4b shows the FRP feature, cprom (cluster prominence). Figure 4c–e show frequency-domain and graphical HRV features. The feature values changed during slow-paced breathing compared to spontaneous breathing. The obtained feature set has non-linear, image-based, frequency-domain, and graphical features. The sample entropy and normalized spectral power of the HF band decreased during slow-paced breathing. Furthermore, cluster prominence, LF/HF ratio, and SD2 increased.

First, the feature set of relevant and non-redundant features was used for classification using LDA, SVM, MLP, IBK (k = 3), DT, and RF classifiers. The classification performance parameters are shown in Table 5. Accuracy is the proportion of accurately categorized instances to the total instance. This study used a 10-fold cross-validation scheme to compute the model. It is clear from the results that classification by relevant and non-redundant features showed the highest accuracy of 96.66 in the SVM classifier with five features.

**Figure 5:**
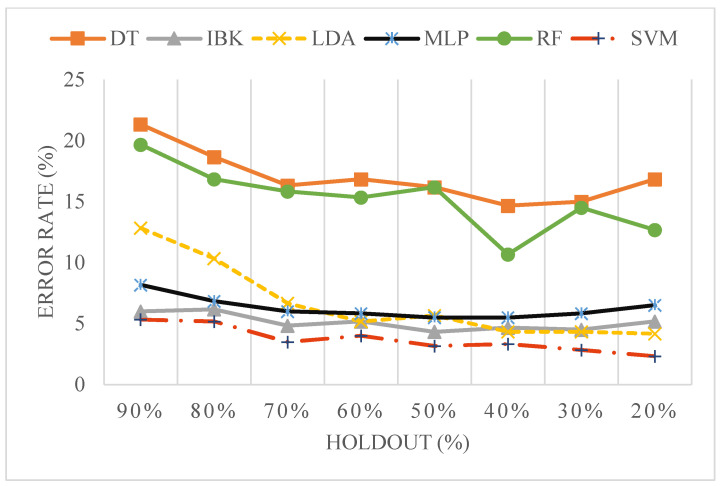
Learning curve analysis of classifiers using five relevant and non-redundant combinations of features across varying holdout proportions.

To further investigate how classification performance varies with the size of the training data, a learning curve was generated using the relevant and non-redundant features, as shown in Figure 5 and detailed in Table S8. The training data proportion was varied systematically using holdout splits from 90% to 10%. Error rates decreased consistently as the training size increased. Among all classifiers, SVM showed the most consistent improvement, with the error rate reducing from 13.33% at 90% holdout to 2.33% at 10% holdout.

In our study, we initially utilized five relevant and non-redundant features for classification. However, the number of features remained relatively high, prompting us to explore the potential for a more refined feature subset. To optimize this, we employed a greedy stepwise forward feature subset evaluator. The results, as detailed in Table 6, reveal that the most effective classification was achieved using a subset of three features, leading to an impressive accuracy of 96.66% with the SVM classifier. Notably, this optimal subset comprised cluster prominence (a key FRP feature) with an FDR of 1.256, LF/HF ratio (a frequency-domain feature) with an FDR of 1.044, and SD2 (a graphical feature) with an FDR of 0.989.

**Figure 6:**
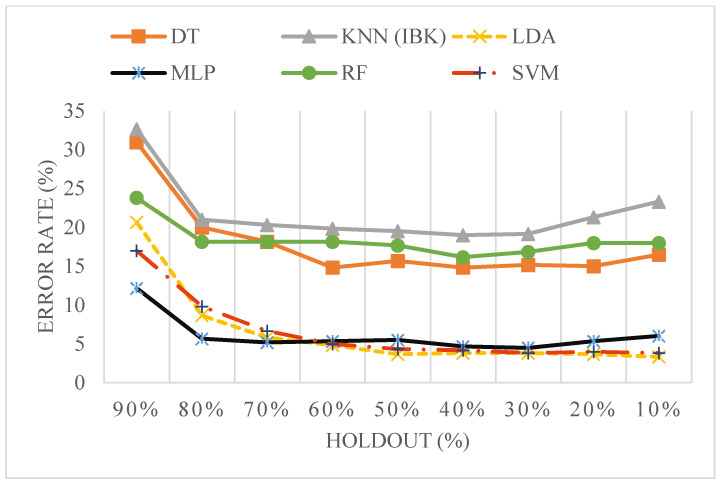
Error rate trends with increasing training data proportions for six classifiers following greedy stepwise feature selection.

To further assess the robustness of the classifiers, a learning curve analysis was conducted by varying the holdout percentage, starting from 90% holdout (10% training data) and reducing it incrementally to 10% holdout (90% training data), as shown in Table S9. The corresponding error rates were plotted for each classifier—decision tree (DT), k-nearest neighbour (IBK), linear discriminant analysis (LDA), multi-layer perceptron (MLP), random forest (RF), and support vector machine (SVM)—as shown in Figure 6.

The plot reveals a general decrease in error rate as the holdout percentage decreases, indicating improved model performance with increased training data. Classifiers like LDA, MLP, and SVM show sharp improvements and lower error rates, while DT and IBK exhibit comparatively higher error levels across holdout variations.

The high classification performance observed in the learning curve analysis, particularly with SVM using a refined subset of features, reaffirms the effectiveness of our feature selection approach. Among these, the persistent contribution of the image-based FRP feature (cluster prominence) across multiple classifiers emphasizes its diagnostic importance.

The high accuracy and the exceptional FDR values of the FRP features underscore the breakthrough achieved through texture-based analysis. This approach not only showcased the FRP method’s ability to effectively differentiate between spontaneous and slow-paced breathing but also highlighted its novelty and effectiveness. The high discriminative power of FRP features suggests significant real-world applications, particularly in wearable health devices designed for stress management. By providing precise, real-time feedback on relaxation states, FRP image-based methods can enhance the practice and monitoring of relaxation techniques, aligning perfectly with our initial motivation to offer objective, data-driven insights for improved stress management and physiological awareness.

## 4. Discussion

Slow-paced breathing is a highly effective method for inducing relaxation, and our study provides compelling evidence to support this. Our results show that HRV features change significantly during slow-paced breathing, corroborating previous findings. Specifically, we observed significant increases in time-domain and graphical features such as the Root Mean Square of Successive Differences (RMSSD) and SD1. These changes reflect higher HRV time series amplitudes under slow-paced breathing, aligning with established physiological markers of relaxation [47,48]. The RBC values for RMSSD (+0.5828) and SD1 (+0.5828) indicate moderate-to-strong positive effects, supporting a consistent increase in these features across participants and reinforcing their role as reliable indicators of parasympathetic activation.

Interestingly, in this research, the spectral measure of normalized LF spectral band power increased during slow-paced breathing, consistent with earlier studies [18]. This effect is due to the coupling relationship between the cardiovascular and respiratory systems becoming prominent at low respiration frequency. Since the respiration frequency during slow-paced breathing overlaps with the LF band frequency range, the increase in LFnu is expected. Although LFnu often reflects sympathetic activity in stress, here its strong increase (RBC = +0.9957) likely indicates vagal activation from slow-paced breathing.

Similarly, the LF_HF ratio increased significantly, with a very strong RBC of +0.9957. This shows that LF power rose more than HF power. While LF_HF is often seen as a stress marker, in this context, the rise reflects autonomic system response to slow-paced breathing, not stress.

Overall, the frequency-domain features not only showed statistically significant changes, but also demonstrated very strong and consistent effect sizes, supporting the interpretation that slow-paced breathing induces a robust autonomic shift favouring relaxation.

Graphical features like SD1 and SD2, which indicate long-term and short-term variability in HRV time series [18,49], demonstrated significant changes. In particular, SD2 increased notably, as seen in Figure 4e. This suggests enhanced variability during slow-paced breathing. The effect size, captured by the rank–biserial correlation (RBC), was very strong (+0.9742) for SD2, confirming a consistent and substantial increase across participants.

While graphical features like SD1 and SD2 provide valuable insights into long-term and short-term HRV, the complexity of the HRV time series during slow-paced breathing necessitates exploring non-linear analysis. Given the non-stationary nature of HRV time series, time-domain and frequency-domain features alone may not capture all variations, particularly those driven by breathing patterns [50]. This is where non-linear entropy measures offer a deeper understanding. Therefore, in this study, we computed non-linear entropy features. The non-linear time-series analysis methods require stationary and long data lengths [51]. Interestingly, all the entropy measures change during slow-paced breathing. Slow-paced breathing reduced sample entropy, implying that the HRV time series is less complex [52]. Previous research suggested that sample and fuzzy entropy values decrease when the signal frequency decreases [53]. We observed from frequency-domain analysis that during slow-paced breathing, the HRV time series frequency became prominent in the LF band; therefore, this could be a possible reason for the decrease in the entropy measure.

Although traditional HRV features such as LF/HF ratio and SD2 have been widely studied, our use of the FRP image-based method introduces a novel approach to HRV classification. By converting HRV time-series data into images and applying texture analysis techniques, we identified unique features, such as cluster prominence, that enhance discriminatory power, particularly during slow-paced breathing. The value of the feature increased significantly during slow-paced breathing. Notably, cluster prominence showed both a high Fisher discriminant ratio (FDR) of 1.256 and a very strong positive rank–biserial correlation (RBC) of +0.9871, indicating a consistent and substantial increase in this feature’s value during slow-paced breathing. Cluster prominence indicates the skewness and asymmetry of the GLCM. A higher value of cluster prominence implies more asymmetry about the mean. Here, the FRP image of the HRV time series during slow-paced breathing indicates the high variation in grey scale values throughout the image. This could be the reason for the significant increase in the cluster prominence value.

While each domain of HRV feature extraction, whether time-domain, frequency-domain, image-based, or graphical, provides useful indicators, an extensive feature set can introduce redundancy and reduce computational efficiency. Therefore, to refine our approach, we applied feature selection and reduction methods to isolate only the most relevant and non-redundant features, ensuring that the final model is accurate and practical for real-time applications. In this study, the FDR value was used to sort the features. The higher FDR value implies that the feature has more discriminating strength and is relatively significant. Table 3 shows the thirteen relevant features obtained after applying FDR (>0.95) selection. Eight FRP features have FDRs above 0.95; clearly, the FRP image-based method provides more relevant features with higher discriminant power. Table 4 shows the list of pertinent (FDR > 0.95) and non-redundant features obtained after applying the correlation matrix reduction technique. The five relevant and non-redundant features belong to frequency-domain, graphical, non-linear, and image-based methods. Importantly, each of these features demonstrated strong to very strong RBC values, confirming their consistent and meaningful changes during slow-paced breathing. Interestingly, out of eight FRP features, only one FRP feature (cluster prominence) was left after feature reduction. It could be implied from this observation that FRP features are highly correlated. Further, the relevant and non-redundant features that were obtained are used in classification. The highest classification accuracy of 96.66% is achieved using all five features with an SVM classifier. The results indicate that different feature extraction methods have a significant and unique contribution to classification.

In any classification problem, dimensionality reduction leads to better computation performance in real-time applications. Here, we applied the greedy search method on the previously obtained feature set to extract the feature subset. Interestingly, further investigation to reduce the number of features revealed the importance of the FRP feature (cluster prominence). The cluster prominence feature was common in LDA, SVM, MLP, and DT classifiers. Classifiers trained with cluster prominence as a feature demonstrate higher specificity, as shown in Table 6. One possible reason for this observation is the highest FDR value of cluster prominence among the feature subsets. Furthermore, specificity is considered a critical classifier performance parameter in medical diagnosis. It is the proportion of positives incorrectly identified as negatives. In other words, it indicates the misrepresentation of data, which could have profound implications. Surprisingly, our result showed 100% specificity and was highest in the case of feature subsets with cluster prominence as a feature. This feature subset handles misidentification relatively better.

To assess model stability across varying data sizes, we performed a comprehensive learning curve analysis using both the initial five-feature set and the reduced greedy-selected subset as shown in Figure 5 and Figure 6, respectively. The support vector machine (SVM) classifier consistently demonstrated superior performance across all holdout levels. Specifically, the error rate decreased steadily as the training data increased, reaching an accuracy of 97.67% at 10% holdout (i.e., 90% training data). This performance slightly exceeded the cross-validation accuracy of 96.66%, indicating that the model continues to benefit from increased training data without signs of overfitting.

What is particularly significant is that after applying greedy stepwise feature selection, which reduced the number of features to just three, the SVM classifier’s performance remained robust. At the 10% holdout level, the model still achieved 96.17% accuracy, nearly identical to its cross-validation performance. This convergence strongly suggests that the selected feature subset retains sufficient discriminative power for reliable classification, even with fewer features.

**Figure 7:**
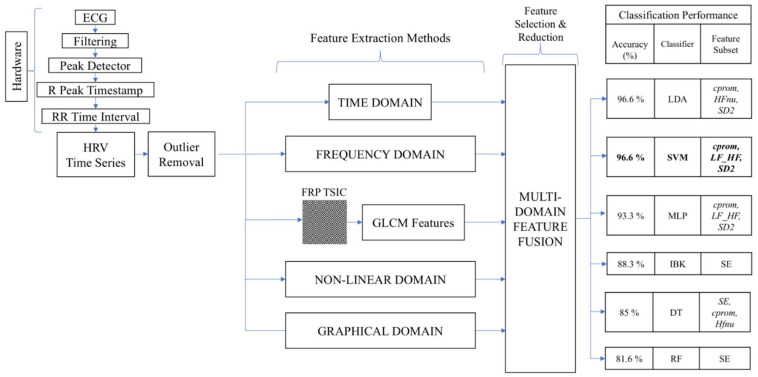
Schematic illustration of the best-performing classifier and multi-domain feature subset. Comparison of all six classifiers, including their classification accuracy and associated feature subsets. The best-performing classifier (SVM with 96.6% accuracy) and its three-feature subset are highlighted in bold.

Our findings have important practical implications. The consistency between cross-validation and holdout-based learning curves highlights the model’s generalization capability and supports the effectiveness of feature reduction for real-world applications where computational efficiency and stability are critical. The high classification accuracy achieved with the SVM classifier, particularly using cluster prominence as a key feature, underscores the potential for FRP image-based methods to enhance real-time monitoring of relaxation states, as shown in Figure 7. Wearable devices leveraging these methods could provide users with precise, objective feedback on their relaxation states, improving the effectiveness of relaxation techniques and stress management strategies.

This research directly supports our initial motivation of providing objective, data-driven feedback to improve relaxation practices. By overcoming the limitations of subjective assessment, our study delivers a robust, evidence-based framework for refining and monitoring relaxation techniques. The capability to offer accurate, real-time feedback on relaxation states not only enhances user engagement but also improves the overall effectiveness of stress management interventions. Importantly, converting the HRV time series into image representations—particularly through fuzzy recurrence plots—adds a powerful visual dimension to this framework. These images make the subtle physiological changes associated with relaxation clearly visible, enabling both clinicians and individuals to easily observe and track the body’s response to slow-paced breathing. This visual insight supports better interpretation, communication, and adherence in relaxation-based therapies.

While deep learning-based classifiers, such as convolutional neural networks (CNNs), have shown strong performance in various ECG-based classification tasks, particularly in pathological detection scenarios, their application in physiological modulation studies presents certain limitations. Deep learning models often rely on large annotated datasets to generalize effectively and may primarily extract morphological features from raw ECG signals, which are less informative in healthy subjects undergoing controlled breathing. In contrast, our approach leverages domain knowledge of HRV dynamics and transforms RR interval time series into fuzzy recurrence plots, enabling the capture of subtle, nonstationary autonomic fluctuations that may not manifest as distinct morphological patterns in ECG waveforms. Furthermore, our method allows for interpretable feature extraction across multiple domains (time, frequency, non-linear, and texture-based features), facilitating physiological insight that is often challenging to obtain from deep learning models, which generally act as black-box classifiers. Nevertheless, we acknowledge that deep learning models may offer advantages in cases where sufficiently large, diverse datasets are available or where morphological abnormalities are prominent.

From a practical implementation perspective, the proposed method demonstrates promising feasibility for real-time deployment in resource-constrained wearable systems. The SVM classifier, which demonstrated the best performance in our study using only three selected features, is computationally lightweight and well-suited for real-time implementation in embedded processors commonly used in wearable devices. This stands in contrast to deep learning models that often require substantial processing resources and large annotated datasets. Furthermore, HRV analysis is inherently based on RR interval time series, which have a much lower sampling rate compared to full ECG signals, further reducing data processing requirements. While the generation of fuzzy recurrence plots involves fuzzy clustering, this step is applied to RR intervals rather than high-frequency ECG signals, making the computation manageable for modern low-power devices with appropriate optimization. Therefore, the overall framework holds strong potential for integration into wearable platforms to enable real-time monitoring of relaxation states.

However, there are some methodological limitations in the current study that warrant further investigation. While this study evaluated the effects of slow-paced breathing at a rate of four breaths per minute, it is crucial to explore other breathing frequencies, particularly the individual’s resonance frequency. The resonance frequency is where heart rate variability (HRV) reaches its peak and the relaxation response is most pronounced. Since this optimal frequency may shift with regular practice, it is essential to assess and adjust the breathing rate over time for individualized applications. Additionally, the participants in this study were between 18 and 31 years old, representing a young demographic. Future research should include broader age groups, such as minors, middle-aged adults, and the elderly, to better understand how slow-paced breathing affects individuals across the lifespan. In this context, it is important to acknowledge that HRV characteristics exhibit significant age-related variability due to physiological changes in autonomic function, the presence of lifestyle-related diseases, and the increased prevalence of chronic or pathological conditions in older individuals. These factors can potentially confound the interpretation of relaxation-induced autonomic modulation observed during slow-paced breathing. Since the primary objective of this study was to assess the autonomic response in healthy individuals under controlled breathing conditions, we restricted our participant group to a younger, healthy demographic to minimize these confounding influences. Nonetheless, future studies incorporating broader age ranges and clinical populations would be valuable to evaluate the generalizability and clinical applicability of the proposed methodology. Data acquisition posed a challenge, and the sample size was limited to 60 participants, which may restrict the generalizability of the findings. Larger, more diverse sample sizes in future studies will allow for more statistically robust and significant results, offering deeper insights into the broader applicability of slow-paced breathing interventions.

While these limitations highlight areas for further exploration, it is also essential to consider the methodological implications of data acquisition and processing in the current study. One critical factor is the need for accurate peak detection in ECG signals, as any failure in proper peak identification can introduce outliers into the HRV time series, potentially distorting the FRP image texture and leading to inaccurate feature extraction. To address this, we implemented a threshold-based outlier removal technique, which improved the reliability of the HRV series and enhanced the consistency of feature computation.

Additionally, in the FRP image generation process, the number of fuzzy clusters represents an important parameter influencing texture representation. The cluster number was selected based on preliminary empirical evaluations aimed at balancing texture detail with computational feasibility. Excessively high cluster numbers may introduce noise or over-segmentation, while having too few clusters risks underrepresenting the inherent variability in RR interval dynamics. The chosen cluster size effectively captured the nonstationary and quasi-periodic patterns associated with respiration-induced HRV modulation while maintaining computational efficiency. Nonetheless, we acknowledge that a more systematic sensitivity analysis of fuzzy clustering parameters could offer further insights and will be considered in future investigations.

## 5. Conclusions

This study presents a novel and practical approach for monitoring relaxation states by combining visual and automated analysis of HRV time series. By converting HRV data into fuzzy recurrence plots (FRPs), we introduced a visually interpretable modality that reveals clear texture-based differences between spontaneous and slow-paced breathing. These image-based representations make physiological changes, such as those driven by respiratory sinus arrhythmia, accessible not only to clinicians, but also to users themselves, enabling intuitive and quick tracking of relaxation.

To complement this interpretability with robust automation, we developed a multi-domain feature fusion framework. Features from five domains—time, frequency, non-linear, geometric, and image-based—were extracted and refined using statistical selection techniques. The final model achieved a classification accuracy of 96.6% with only three optimal features: cluster prominence, LF/HF ratio, and SD2. This balance between high accuracy and minimal computational demand is essential for real-time monitoring in wearable devices.

Importantly, our findings address the core challenge of relaxation training: providing objective, real-time feedback. The integration of FRP image-based visualization and machine learning enables a deeper understanding of physiological state transitions, making the system both a diagnostic tool and an aid for biofeedback-driven stress management. The methodology supports the development of efficient, low-power wearable systems that can personalize relaxation practices and improve health outcomes through continuous, real-time monitoring.

## Figures and Tables

**Table 1:** Selected hyperparameters for various entropy calculations.

S. No	Entropy	Hyperparameters
1	Approximate entropy	m = 2, r = 0.2
2	Sample entropy	m = 2, r = 0.2, tau = 1
3	Fuzzy entropy	m = 2, membership function = trapezoidal, tau= 2
4	Amplitude-aware permutation entropy	m = 6, tau = 1, a = 0.5
5	Bubble entropy	m = 10

**Table 2:** Hyperparameters used in classifier methods.

S. No	Classifier	Hyper-Parameters
1	Support vector machine (SVM)	Kernel = radial basis function
2	Linear discriminant analysis (LDA)	R = 1.0 × 10^−6^
3	K-nearest neighbour (IBK)	K = 3
4	C4.5	Confidence factor = 0.25, minimum number of instances = 2
5	Multi-layer perceptron (MLP)	Learning rate = 0.3, momentum = 0.2, number of excerpts= 500
6	Random forest (RF)	K = 0, M = 1, P = 100, I = 100

**Table 3:** HRV features with high discriminatory strength based on FDR analysis.

S No.	Features	FDR Value > 0.95
1	SampEn_DS	1.280185
2	Cluster Prominence *	1.255806
3	Entropy *	1.146591
4	HFnu	1.132644
5	LFnu	1.132644
6	Sum Entropy *	1.110963
7	Energy *	1.084762
8	Information measure of correlation 1 *	1.05102
9	LF_HF	1.043894
10	Maximum probability *	1.015278
11	Homogeneity *	1.009838
12	Difference Entropy *	1.003695
13	SD2	0.989185

This table lists heart rate variability (HRV) features that have a Fisher discrimination ratio (FDR) value greater than 0.95, indicating high discriminatory relevance. The FDR values were computed for all extracted features, and only those exceeding the 0.95 threshold are included here. These features demonstrate strong potential for distinguishing between slow-paced breathing and spontaneous breathing. Features marked with an asterisk (*) represent texture-based features.

**Table 4:** Non-redundant HRV feature set spanning multiple domains.

S. No.	Features	FDR Value	RBC
1	Sample Entropy	1.28	−1
2	Cluster Prominence	1.256	0.987
3	HFnu	1.133	−0.996
4	LF_HF	1.044	0.996
5	SD2	0.989	0.974

This table displays the feature set of five non-redundant HRV features obtained after applying correlation-based redundancy reduction to the FDR-selected features. The retained features span multiple domains—non-linear, image-based, frequency, and graphical—demonstrating a diverse, yet compact, representation of the most relevant features. Additionally, the table includes the corresponding rank–biserial correlation (RBC) values to quantify the effect size and further validate the discriminative power of these features.

**Table 5:** Classifier performance with five relevant and non-redundant combinations of features.

Classifier	ACC%	SEN%	SPE%	F1-SCORE	AUC%
LDA	95	93.3	96.6	0.95	96.6
**SVM**	**96.6**	**93.3**	**100**	**0.967**	**96.7**
MLP	93.3	90	96.7	0.933	96.4
IBK	95	93.3	96.6	0.95	97.6
DT	85	80	90	0.85	88.4
RF	90	90	90	0.9	95.9

**Table 6:** Classifier performance using greedy stepwise optimized feature subsets.

Classifier	ACC	SEN	SPE	F1-SCORE	AUC	Feature Subset
LDA ^#^	96.6	96.7	96.7	0.967	96.4	cprom, HFnu, SD2
**SVM ^#^**	**96.6**	**93.3**	**100**	**0.967**	**96.7**	**cprom, LF_HF, SD2**
MLP ^#^	93.3	90	96.7	0.933	96.2	cprom, LF_HF, SD2
IBK	88.3	90	86.7	0.850	90.9	SE
DT ^#^	85	80	90	0.883	88.4	SE, cprom, HFnu
RF	81.6	83.3	80	0.817	90.6	SE

This table shows the performance of each classifier when trained on a feature subset selected through greedy stepwise optimization, aiming to maximize classification accuracy. The final column lists the optimized feature subset for each classifier. Features are abbreviated (e.g., cprom for cluster prominence, SE for sample entropy). The symbol # indicates classifiers that share cluster prominence (cprom) as a common feature in their optimal subset, highlighting its significance across multiple models.

## Data Availability

The raw data supporting the conclusions of this article will be made available by the authors on request.

## Supplementary Materials

The following supporting information can be downloaded at: https://www.mdpi.com/article/10.3390/s25134210/s1, Table S1: Standard HRV features and description; Table S2: GLCM features extracted from each FRP image; Table S3: Interpretation guide for rank–biserial correlation (RBC) effect sizes; Table S4: Mean and standard deviation of standard HRV features under spontaneous breathing and slow-paced breathing with FDR value; Table S5: Mean and standard deviation of non-linear entropy-based HRV features under spontaneous breathing and slow-paced breathing with FDR value; Table S6: Mean and standard deviation of FRP image-based HRV features under spontaneous breathing and slow-paced breathing, with tabulated FDR values; Table S7: Feature-wise rank–biserial correlation (RBC) interpretation; Table S8: Learning curve results using five relevant and non-redundant combinations of features; Table S9: Learning curve results after greedy stepwise feature selection.

## Abbreviations

The following abbreviations are used in this manuscript:

ECG	Electrocardiograms
HRV	Heart rate variability
FRP	Fuzzy recurrence plot
SVM	Support vector machine
ANS	Autonomic nervous system
PNS	Parasympathetic nervous system
SNS	Sympathetic nervous system
HR	Heart rate
RSA	Respiratory sinus arrhythmia
FDR	Fisher discriminant ratio
GLCM	Grey level co-variance matrix
RBC	Rank–biserial correlation
