# Supplementary material for: Visualizing Relaxation in Wearables: Multi-Domain Feature Fusion of HRV Using Fuzzy Recurrence Plots
Source: Sensors (Basel). 2025 Jul 6;25(13):4210. doi: 10.3390/s25134210 (PMC12252487; doi:10.3390/s25134210)
Supplement: Supplementary file 1 [file sensors-25-04210-s001.zip › sensors-3706402-supplementary.pdf]

Table S1: Standard HRV Features and Description

S No	Features	Description of feature	Interpretation	references
		**Time Domain**		
1	Mean RRi	The mean of RR intervals	Central value of RR interval time series, reduced under sympathetic activation and parasympathetic withdrawal	[1]
2	Mean HR	The mean Heart Rate	Central value of Heart rate increases under sympathetic activation and parasympathetic withdrawal	[1]
3	SDNN	Standard deviation of RR intervals	reflects the over-all (both short-term and long-term) variation within the RR-interval series	[2]
4	RMSSD	Square root of the mean squared differences between successive RR intervals	measure of the short-term variability	[2]
5	NN50	Number of successive RR interval pairs that differ more than 50 ms	short-term variation estimate high frequency variations in heart rate	[3]
6	pNN50	NN50 divided by the total number of RR intervals	short-term variation estimate high frequency variations in heart rate	[3]
		**Frequency Domain**		
7	Total Power	Total spectral power	The variance of RR interval	[3]
8	VLF	VLF spectral band power	strongly associated with all-cause mortality	[4]
9	LF	LF spectra band powers	reflects baroreceptor activity during resting conditions, vagal activity during slow respiration rates	[4]
10	HF	HF spectra band powers	respiratory band and reflects parasympathetic activity	[2,4]
11	LF/HF	Ratio between LF and HF band powers	sympathovagal balance under a specific condition (stress and paced breathing relaxation)	[4]
12	LFnu	Powers of LF bands in normalized units	reflects sympathetic activity, vagal under paced breathing	[3]
13	HFnu	Powers of HF bands in normalized units	reflects parasympathetic activity	[3]
		**Graphical**		
14	SD1	Standard deviations of the Poincaré plot along y=x line, ellipse's width	measure short-term variability	[2,4]
15	SD2	Standard deviations of the Poincaré plot along y =x + average RR interval, ellipse's length	measure short term and long-term variability	[2,4]

Standard Heart Rate Variability (HRV) features used in the study. The table lists the feature name, description, physiological interpretation, and relevant references for each feature.

Table S2: GLCM Features Extracted from each FRP Image

S No.	Full name of Features	Features	Formula
1	Autocorrelation	autoc	$F_1 = \sum_{i,j=0}^{n-1} ijC(i,j)$
2	Contrast	contr	$F_2 = \sum_{i,j=0}^{n-1} C(i,j)(i-j)^2$
3	Correlation	corrm	$F_3 = \sum_{i,j=0}^{n-1} C(i,j)\frac{(i-\mu_x)(j-\mu_y)}{\sigma_x\sigma_y}$
4	Cluster prominence	cprom	$F_4 = \sum_{i,j=0}^{n-1} (i-\mu_x+j-\mu_y)^4\, C(i,j)$
5	Cluster Shade	cshad	$F_5 = \sum_{i,j=0}^{n-1} (i-\mu_x+j-\mu_y)^3\, C(i,j)$
6	Dissimilarity	dissi	$F_6 = \sum_{i,j=0}^{n-1} C(i,j)\lvert i-j\rvert$
7	Energy	energ	$F_7 = \sum_{i,j=0}^{n-1} C^2(i,j)$
8	Entropy	entro	$F_8 = -\sum_{i,j=0}^{n-1} C(i,j)\log C(i,j)$
9	Homogeneity	homom	$F_9 = \sum_{i,j=0}^{n-1} \frac{C(i,j)}{1+\lvert i-j\rvert}$
10	Maximum probability	maxpr	$F_{10} = max_{i,j}\, C(i,j)$
11	Sum of sqaures: Variance	sosvh	$F_{11} = -\sum_{i,j=0}^{n-1} (1-\mu)^2 C(i,j)$
12	Sum average	savgh	$F_{12} = \sum_{i=2}^{2N_G} i\, C_{x+y}(i)$
13	Sum variance	svarh	$F_{13} = \sum_{i=2}^{2N_G} (i-F_{14})C_{x+y}(i)$
14	Sum entropy	senth	$F_{14} = -\sum_{i=2}^{2N_G} C_{x+y}(i)\, log\{C_{x+y}(i)\}$
15	Difference variance	dvarh	$F_{15} = variance\ of\ C_{x-y}$
16	Difference entropy	denth	$F_{16} = -\sum_{i=0}^{N_G-1} C_{x+y}(i)\, log\{C_{x-y}(i)\}$
17	Information measure of correlation1	inf1h	$F_{17} = \frac{(F_8 - H_{xy1})}{max\{H_x, H_y\}}$
18	Informaiton measure of correlation2	inf2h	$F_{18} = \left(1-e^{[-2(H_{xy2}-H_{xy})]}\right)^{\frac{1}{2}}$
19	Inverse difference	indnc	$F_{19} = \sum_{i,j=0}^{n-1} \frac{C(i,j)}{1+(i-j)^2}$

Where,

$C_{x+y} = \sum_{i=1}^{G}\sum_{\substack{j=0 \\ i+j=k}}^{G} C(i,j)$, k = 2,3,4….2G and $C_{x-y} = \sum_{i=1}^{G}\sum_{\substack{j=1 \\ \lvert i-j\rvert=k}}^{G} C(i,j)$, k = 0,1,….G-1

$$\mu_x = \sum_{i,j=0}^{n-1} i\, C(i,j) \text{ and } \mu_y = \sum_{i,j=0}^{n-1} j\, C(i,j)$$

$$\sigma_x = \sum_{i,j=0}^{n-1}(i-\mu_x)^2\, C(i,j) \text{ and } \sigma_y = \sum_{i,j=0}^{n-1}(i-\mu_y)^2\, C(i,j)$$

$$S_x(i) = \sum_{j=0}^{n-1} C(i,j) \text{ and } S_y(j) = \sum_{i=0}^{n-1} C(i,j)$$

$$H_{xy1} = -\sum_{i,j=0}^{n-1} C(i,j)\,log\left(S_x(i)S_y(j)\right) \text{ and } H_{xy2} = -\sum_{i,j=0}^{n-1} S_x(i)S_y(j)\,log\left(S_x(i)S_y(j)\right)$$

$$H_x = -\sum_{i=0}^{n-1} S_x(i)\,log\left(S_x(i)\right) \text{ and } H_y = -\sum_{j=0}^{n-1} S_y(j)\,log\left(S_y(j)\right)$$

Gray-Level Co-occurrence Matrix (GLCM) texture features extracted from each Fuzzy Recurrence Plot (FRP) image. The table lists the serial number, full name of each feature, its abbreviation, and the corresponding mathematical formula used for feature extraction.

Table S3: Interpretation Guide for Rank-Biserial Correlation (RBC) Effect Sizes

Rank-Biserial Correlation (RBC) Range	Effect Strength	Interpretation
-1	Perfect Effect	All values changed in one direction
±0.80 to ±0.99	Very Strong Effect	Clear, consistent change
±0.50 to ±0.79	Moderate-to-Strong Effect	Substantial paired change
±0.20 to ±0.49	Small-to-Moderate Effect	Some change, with variability
±0.01 to ±0.19	Weak/Negligible Effect	Minor change, likely not practically relevant
0	No Effect	No direction of change between paired values

This table provides guidance for interpreting the magnitude and direction of Rank-Biserial Correlation (RBC) values derived from paired comparisons. Positive values indicate an increase in the feature post-intervention or condition change, while negative values reflect a decrease. The magnitude denotes the strength of the effect.

Table S4: Mean and Standard Deviation of Standard HRV Features Under Spontaneous Breathing, and Slow-paced Breathing with FDR Value

S No.	Features	Spontaneous Breathing		Slow-Paced Breathing		Trend	FDR	Wilcoxon Sum rank test
		mean	std	mean	std			p-value
1	RMSSD*	35.04	19.18	48.47	22.45	increase	0.579	0.00532
2	SDNN*	49.63	18.14	88.20	32.66	increase	0.949	3.88E-06
3	pNN50	13.91	14.82	16.37	9.48	increase	0.314	0.308615
4	SD1*	24.80	13.58	34.31	15.90	increase	0.579	0.00532
5	SD2*	65.38	22.61	119.68	44.07	increase	1.001	3.18E-06
6	NN50*	54.97	52.30	69.47	36.14	increase	0.403	0.1588
7	Mean RR	745.57	106.63	749.23	98.55	increase	0.133	0.765519
8	Mean HR	81.95	10.79	81.31	9.78	decrease	0.175	0.703564
9	Total Power*	2139.43	1634.77	7605.56	6469.16	increase	0.816	2.88E-06
10	VLF*	494.73	429.60	623.40	338.57	increase	0.409	0.089718
11	LF*	918.77	652.68	6201.65	5693.57	increase	0.881	1.73E-06
12	HF	725.94	969.55	780.50	673.93	increase	0.180	0.926255
13	LF/HF*	2.18	1.65	10.70	7.01	increase	1.044	1.92E-06
14	LFnu*	61.46	16.77	87.01	9.50	increase	1.133	1.92E-06
15	HFnu*	38.54	16.77	12.99	9.50	decrease	1.133	1.92E-06

Values represent the mean ± standard deviation of standard HRV features under spontaneous and slow-paced breathing conditions. * indicates statistical significance ($p < 0.05$). "increase" or "decrease" denotes the trend of change in the HRV feature during slow-paced breathing relative to spontaneous breathing. Fisher Discriminant Ratio (FDR) values are included for reference and were used only for feature selection.

Table S5: Mean and Standard Deviation of non-linear Entropy-based HRV features under spontaneous breathing and slow-paced breathing with FDR value

S No.	Features	Spontaneous Breathing		Slow-Paced Breathing		Trend	FDR	Wilcoxon Sum rank test
		mean	std	mean	std			p-value
1	Approximate Entropy	0.0007	0.0031	0.0000	0.0000	decrease	0.413	0.09375
2	Sample* Entropy	1.4672	0.2768	0.8344	0.2959	decrease	1.280	1.73E-06
3	Fuzzy Entropy	2.508457	0.519866	2.3624	0.4775	decrease	0.382	0.071903
4	Amplitude aware Permutation Entropy	1.9714	0.1392	1.9153	0.2009	decrease	0.512	0.271155
5	Bubble Entropy*	0.5466	0.1877	0.9346	0.4430	increase	0.808	0.001593

Values represent the mean ± standard deviation of Entropy-based HRV features under spontaneous and slow-paced breathing conditions. * indicates statistical significance ($p < 0.05$). "increase" or "decrease" denotes the trend of change in the HRV feature during slow-paced breathing relative to spontaneous breathing. Fisher Discriminant Ratio (FDR) values are included for reference and were used only for feature selection.

Table S6: Mean and Standard Deviation of FRP image-based HRV Features Under Spontaneous Breathing and Slow-paced Breathing, with Tabulated FDR Value

S No.	Features	Spontaneous Breathing		Slow-Paced Breathing		Trend	FDR	Wilcoxon Sum rank test
		mean	std	mean	std			p-value
1	*Autocorrelation	24.58521	1.493483	25.98716	2.669554	increase	0.581	0.007731
2	*Contrast	1.444859	0.383423	1.069124	0.261708	decrease	0.786	3.11E-05
3	*Correlation	0.882093	0.039165	0.929537	0.022112	increase	0.936	6.34E-06
4	*Cluster prominence	885.5758	151.1743	1282.34	196.5597	increase	1.256	2.13E-06
5	Cluster Shade	8.202336	12.23708	14.89535	26.99837	increase	0.372	0.404835
6	*Dissimilarity	0.630465	0.121118	0.484513	0.087271	decrease	0.893	1.24E-05
7	*Energy	0.070866	0.014467	0.124778	0.038254	increase	1.085	1.92E-06
8	*Entropy	3.173132	0.137239	2.815395	0.215051	decrease	1.147	2.35E-06
9	*Homogeneity	0.78795	0.030786	0.83323	0.024429	increase	1.010	4.73E-06
10	*Maximum probability	0.150792	0.043188	0.245817	0.065885	increase	1.015	3.52E-06
11	*Sum of sqaures: Variance	25.17094	1.489793	26.38846	2.657035	increase	0.541	0.013975
12	Sum average	8.720404	0.368699	8.647185	0.608827	increase	0.222	0.975387

13	*Sum variance	61.93824	4.318776	70.09974	8.390114	increase	0.840	0.000529
14	*Sum entropy	2.53826	0.054141	2.330434	0.144267	decrease	1.111	1.92E-06
15	*Difference variance	1.444859	0.383423	1.069124	0.261708	decrease	0.786	3.11E-05
16	*Difference entropy	1.051283	0.099682	0.899377	0.088875	decrease	1.004	4.73E-06
17	*Information measure of correlation1	-0.43629	0.05158	-0.51613	0.041251	decrease	1.051	3.88E-06
18	*Informaiton measure of correlation2	0.908724	0.01978	0.925664	0.009272	increase	0.789	0.000148
19	*Inverse difference	0.938146	0.010932	0.95209	0.008054	increase	0.925	9.32E-06

Values represent the mean ± standard deviation of FRP_GLCM-based HRV features under spontaneous and slow-paced breathing conditions. * indicates statistical significance ($p < 0.05$). "increase" or "decrease" denotes the trend of change in the HRV feature during slow-paced breathing relative to spontaneous breathing. Fisher Discriminant Ratio (FDR) values are included for reference and were used only for feature selection.

Table S7: Feature-wise Rank-Biserial Correlation (RBC) Interpretation

Feature	RBC	Effect Direction	Effect Strength
RMSSD	0.583	Increase	Moderate to Strong
SDNN	0.966	Increase	Very Strong
pNN50	0.213	Increase	Small to Moderate
SD1	0.583	Increase	Moderate to Strong
SD2	0.974	Increase	Very Strong
NN50	0.295	Increase	Small to Moderate
Mean	0.062	Increase	Weak / Negligible
MeanHR	–0.080	Decrease	Weak / Negligible
TotalPower	0.978	Increase	Very Strong
VLF	0.355	Increase	Small to Moderate
LF	1	Increase	Very Strong (Perfect)
HF	0.019	Increase	Weak / Negligible
LF_HF	0.996	Increase	Very Strong
LFnu	0.996	Increase	Very Strong
HFnu	–0.996	Decrease	Very Strong
AmplitudeAware_PE	–0.376	Decrease	Small to Moderate
ApEn	–0.810	Decrease	Very Strong
SampEn_DS	–1.000	Decrease	Very Strong (Perfect)
BubbleEntropy_BE	–0.957	Decrease	Very Strong
FM_2	–0.484	Decrease	Moderate to Strong
autoc	0.535	Increase	Moderate to Strong
contr	–0.849	Decrease	Very Strong
corrm	0.935	Increase	Very Strong
cprom	0.987	Increase	Very Strong
cshad	0.144	Increase	Weak / Negligible

dissi	–0.910	Decrease	Very Strong
energ	0.996	Increase	Very Strong
entro	–1.000	Decrease	Very Strong (Perfect)
homom	0.948	Increase	Very Strong
maxpr	0.953	Increase	Very Strong
sosvh	0.488	Increase	Small to Moderate
savgh	–0.028	Decrease	Weak / Negligible
svarh	0.742	Increase	Moderate to Strong
senth	–0.996	Decrease	Very Strong
dvarh	–0.849	Decrease	Very Strong
denth	–0.957	Decrease	Very Strong
inf1h	–0.966	Decrease	Very Strong
inf2h	0.781	Increase	Moderate to Strong
indnc	0.927	Increase	Very Strong

This table summarizes the direction and strength of change for each feature based on Rank-Biserial Correlation (RBC) values. Positive RBC values indicate higher values in the post-condition (e.g., after intervention), while negative values indicate lower values. The magnitude reflects the effect size strength, categorized using the interpretation guide in Table S3.

Table S8: Learning Curve Results Using Five Relevant and Non-redundant Combinations of Features

Holdout %	DT	IBK	LDA	MLP	RF	SVM
90%	30.83	21.33	22.67	11.83	21.67	13.33
80%	21.33	6	12.83	8.17	19.67	5.33
70%	18.67	6.17	10.33	6.83	16.83	5.17
60%	16.33	4.83	6.67	6	15.83	3.5
50%	16.83	5.17	5.17	5.83	15.33	4
40%	16.17	4.33	5.67	5.5	16.17	3.17
30%	14.67	4.67	4.33	5.5	10.67	3.33
20%	15	4.5	4.33	5.83	14.5	2.83
10%	16.83	5.17	4.17	6.5	12.67	2.33

Classification error rates (%) across varying holdout proportions (90% to 10%) using the initial set of relevant and non-redundant features. Results are reported for six classifiers: DT, IBK, LDA, MLP, RF, and SVM. A general trend of decreasing error rate is observed as training data increases, highlighting improved generalization performance.

Table S9: Learning Curve Results After Greedy Stepwise Feature Selection

Holdout %	DT	IBK	LDA	MLP	RF	SVM
90%	31	32.67	20.67	12.17	23.83	17
80%	20	21	8.67	5.67	18.17	9.83
70%	18.17	20.33	5.83	5.17	18.17	6.67
60%	14.83	19.83	4.83	5.33	18.17	5
50%	15.67	19.5	3.67	5.5	17.67	4.33
40%	14.83	19	3.83	4.67	16.17	4.17

30%	15.17	19.17	3.83	4.5	16.83	3.83
20%	15	21.33	3.67	5.33	18	4
10%	16.5	23.33	3.33	6	18	3.83

Classification error rates (%) across varying holdout proportions (90% to 10%) using an optimized feature subset obtained through Greedy Stepwise selection. Compared to the initial feature set, some classifiers—especially SVM—maintain or improve performance with fewer features, demonstrating effective dimensionality reduction without performance loss.

References

1. Salahuddin, L.; Cho, J.; Jeong, M.G.; Kim, D. Ultra Short Term Analysis of Heart Rate Variability for Monitoring Mental Stress in Mobile Settings. *Annual International Conference of the IEEE Engineering in Medicine and Biology - Proceedings* 2007, 4656–4659, doi:10.1109/IEMBS.2007.4353378.
2. Tarvainen, M.P.; Niskanen, J.P.; Lipponen, J.A.; Ranta-aho, P.O.; Karjalainen, P.A. Kubios HRV - Heart Rate Variability Analysis Software. *Comput Methods Programs Biomed* 2014, *113*, 210–220, doi:10.1016/j.cmpb.2013.07.024.
3. Heart Rate Variability: Standards of Measurement, Physiological Interpretation and Clinical Use. Task Force of the European Society of Cardiology and the North American Society of Pacing and Electrophysiology. *Circulation* 1996, *93*, 1043–1065.
4. Shaffer, F.; Ginsberg, J.P. An Overview of Heart Rate Variability Metrics and Norms. *Frontiers in Public Health* 2017, *5*, 1–17.
